# Hypoglycemic agents and incidence of pancreatic cancer in diabetic patients: a meta-analysis

**DOI:** 10.3389/fphar.2023.1193610

**Published:** 2023-07-11

**Authors:** Zimo Zhao, Xinyi He, Yan Sun

**Affiliations:** ^1^ First Clinical Medical College, China Medical University, Shenyang, China; ^2^ Clinical Department I, China Medical University, Shenyang, China; ^3^ Department of Gastroenterology, Shengjing Hospital of China Medical University, Shenyang, China

**Keywords:** hypoglycemic agents, metformin, sulfonylureas, thiazolidinediones, insulin, pancreatic cancer, diabetes mellitus

## Abstract

**Background and aims:** Hypoglycemic agents are the primary therapeutic approach for the treatment of diabetes and have been postulated to impact pancreatic cancer (PC) incidence in diabetic patients. We conducted a meta-analysis to further evaluate and establish the associations between four common types of hypoglycemic agents [metformin, sulfonylureas, thiazolidinediones (TZDs), and insulin] and PC incidence in individuals with diabetes mellitus (DM).

**Methods:** A comprehensive literature search of PubMed, Web of Science, Embase, and the Cochrane Library identified studies that analyzed the relationship between hypoglycemic agents and PC published between January 2012 and September 2022. Randomized control trials (RCTs), cohorts, and case–control studies were included if there was clear and evaluated defined exposure to the involved hypoglycemic agents and reported PC outcomes in patients with DM. Furthermore, reported relative risks or odds ratios (ORs) or other provided data were required for the calculation of odds ratios. Summary odds ratio estimates with a 95% confidence interval (CI) were estimated using the random-effects model. Additionally, subgroup analysis was performed to figure out the source of heterogeneity. Sensitivity analysis and publication bias detection were also performed.

**Results:** A total of 11 studies were identified that evaluated one or more of the hypoglycemic agents, including three case–control studies and eight cohort studies. Among these, nine focused on metformin, six on sulfonylureas, seven on TZDs, and seven on insulin. Meta-analysis of the 11 observational studies reported no significant association between metformin (OR = 1.04, 95% CI 0.73–1.46) or TZDs (OR = 1.13, 95% CI 0.73–1.75) and PC incidence, while the risk of PC increased by 79% and 185% with sulfonylureas (OR = 1.79, 95% CI 1.29–2.49) and insulin (OR = 2.85, 95% CI 1.75–4.64), respectively. Considerable heterogeneity was observed among the studies and could not be fully accounted for by study design, region, or adjustment for other hypoglycemic agents.

**Conclusion:** Sulfonylureas and insulin may increase the incidence of pancreatic cancer in diabetic patients, with varying effects observed among different ethnicities (Asian and Western). Due to significant heterogeneity across studies, further interpretation of the relationship between hypoglycemic agents and pancreatic cancer incidence in diabetic patients requires well-adjusted data and better-organized clinical trials.

## Introduction

Pancreatic cancer (PC) is a highly challenging gastrointestinal oncology malignancy with the poorest prognosis, which remains refractory to treatment and ranks the fourth leading cause of cancer-related mortality worldwide (Aier et al., 2019). The incidence and mortality rates of PC are on the rise in various populations, particularly among women as well as younger and older individuals (Huang et al., 2021). According to epidemiological studies, numerous risk factors have been identified as potential causes of pancreatic cancer, including but not limited to smoking, diabetes mellitus (DM), alcohol consumption, obesity, and a family history of the disease (Aier et al., 2019).

Among the risk factors, the most harmful risk factor is smoking (Klein, 2021). Another crucial factor is DM, which is deemed a significant risk factor for the development of PC. The relationship between DM and PC is multifaceted, with epidemiological evidence indicating a direct association between type 2 diabetes and PC, while also supporting an inverse relationship with DM duration (Everhart and Wright, 1995; Pannala et al., 2009; Giovannucci et al., 2010; Ben et al., 2011; McAuliffe and Christein, 2013; Haugvik et al., 2015; Carreras-Torres et al., 2017; Sharma et al., 2018; Pearson-Stuttard et al., 2021). Long-standing DM is a predisposing factor for PC development, while new-onset DM may be either a consequence or an early manifestation of PC (Andersen et al., 2017; Pizzato et al., 2019).

Hypoglycemic agents are crucial components of the treatment regimen for patients with DM, and it is widely believed that they may have an impact on the incidence of PC in this population. Previous epidemiological and preclinical investigations have suggested that insulin may promote the development of pancreatic cancer, while metformin has been shown to exert anti-cancer effects through its actions on transformed pancreatic cells (Kim et al., 2020; Eibl and Rozengurt, 2021). Sulfonylureas (SUs) are suggested to accelerate the pancreatic β-cell mass loss via apoptosis, and the results of observational studies show that it may increase the risk of PC. However, both metformin and SU were also reported to have no effect on any sites of cancer, which is confusing and needs further clarification (Bodmer et al., 2012; Soranna et al., 2012; Singh et al., 2013; Szymczak-Pajor et al., 2021). The case is the same for thiazolidinediones (TZDs). Studies suggest that TZDs inhibit the proliferation and metastasis of human PC cells, while it is also shown that TZDs do not have a protective or harmful effect (Singh et al., 2013; Ninomiya et al., 2014).

Due to the complicated associations between hypoglycemic agents and PC risk, as well as the poor prognosis of PC, we performed a meta-analysis to further evaluate and clarify the association between the use of hypoglycemic agents (metformin, SU, TZDs, and insulin) and PC incidence in patients with DM.

## Methods

### Search strategy

We searched MEDLINE (National Library of Medicine), Web of Science, Embase (Elsevier), and the Cochrane Library for all relevant studies on hypoglycemic agents and PC incidence in diabetic patients published between January 2012 and September 2022. The Medical Subject Heading (MeSH) terms used in the search are “metformin,” “thiazolidinediones,” “insulin,” “sulfonylurea compounds,” “agents,” “hypoglycemic,” and “pancreatic neoplasms.” Different spellings of the drugs’ names were taken into consideration and included in the search strategy to complete the search results.

### Study selection

Studies included in the meta-analysis were RCTs or observational studies that met the following inclusion criteria: 1) case–control study, cohort study, or RCT; 2) clear and evaluated defined exposure to the involved hypoglycemic agents; 3) reported PC outcome in patients with DM; and 4) reported relative risks or odds ratio (OR) or other provided data for the calculation of OR. In addition, studies were excluded when they were: 1) reviews, case reports, letters, editorials, or commentaries; 2) cancer treatment, prognosis, or mortality studies; 3) unable to extract needed data; and 4) from the same population but the less comprehensive one.

### Data extraction

The data of the studies were extracted independently by two researchers—Zimo Zhao and Xinyi He—to a standardized form. The results were cross-checked, and the differences were solved by consensus. The following data were collected from each study: the first author, year of publication, region, study design, study population, time period, hypoglycemic agents involved, reported primary outcome, frequency, dose and duration of usage (if reported), number of subjects in each group, OR, RR, 95% confidence intervals (CIs), and adjusted variables.

### Quality assessment

The study quality was assessed with the Newcastle–Ottawa Scale (NOS) (Wells et al., 2011) for observational studies by two researchers, Zimo Zhao and Xinyi He, and all discrepancies were discussed and resolved by a third researcher, Yan Sun. The quality of the included studies was evaluated based on three aspects according to the scale: the selection of the study populations, the comparability of the populations, and the ascertainment of exposure.

### Statistical analysis

The random-effects model described by DerSimonian and Laird (1986) was used to calculate summarized OR and 95% CI when quantifying the relationships between the use of hypoglycemic agents and PC risk. As the PC incidence in the general population is relatively low, the RRs (relative risks) and HRs (hazard ratios) were considered an approximation of ORs. The statistical heterogeneity among the studies was assessed with both the Q and I^2^ statistics, and it was considered statistically significant for heterogeneity if the *p*-value was <0.10 or the I^2^ value was >50% (Thompson and Pocock, 1991; Higgins et al., 2003). The causes of heterogeneity were investigated by subgroup analysis based on study design, region, and whether adjusted for other hypoglycemic agents. We conducted sensitivity analysis by omitting one study in turn to check whether any single study would influence the results. Publication bias was assessed using funnel plots and Egger’s test (*p* < 0.10 was considered to indicate significant publication bias) (Egger et al., 1997). Stata 15 (Stata Corporation, College Station, TX) was used for data synthesis and analysis.

## Results

### Study characteristics and quality assessment

**FIGURE 1:**
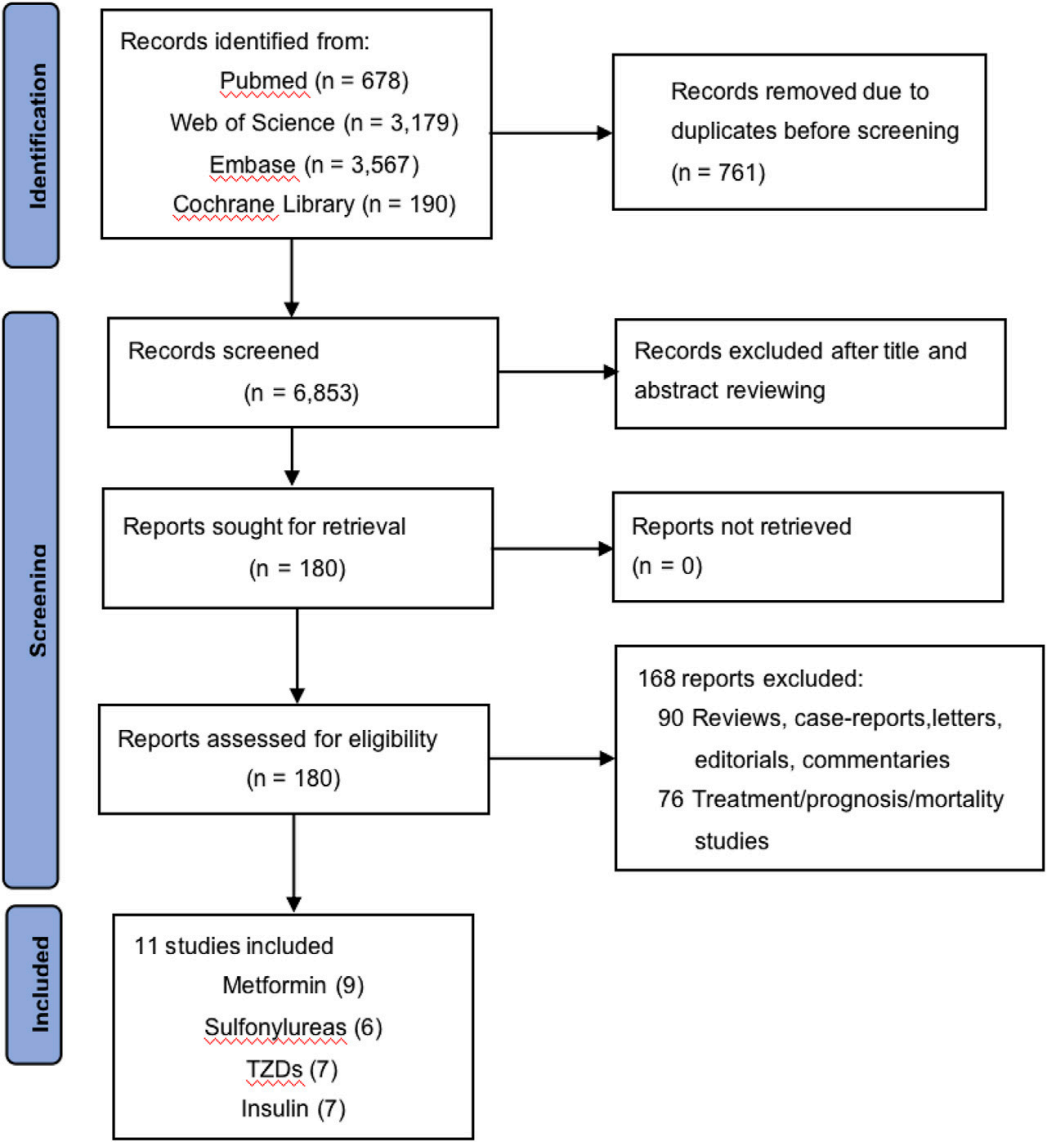
Flow chart of the selection of publications included in this meta-analysis.

The process of study selection is shown in Figure 1. A total of 7,614 records were identified through database searching, and 11 studies finally met the inclusion criteria, including three case–control studies and eight cohort studies, among which nine focused on metformin (Chiu et al., 2013; Funch et al., 2014; Lewis et al., 2015; Lu et al., 2015; Walker et al., 2015; Lee et al., 2018; Tseng, 2018; Lai et al., 2019; Feola et al., 2022), six on SU (Chiu et al., 2013; Funch et al., 2014; Lewis et al., 2015; Lin et al., 2015; Lu et al., 2015; Lee et al., 2018), seven on TZDs (Chiu et al., 2013; Funch et al., 2014; Lewis et al., 2015; Lin et al., 2015; Lu et al., 2015; Walker et al., 2015; Lee et al., 2018), and seven on insulin (Capurso et al., 2013; Chiu et al., 2013; Lewis et al., 2015; Lin et al., 2015; Lu et al., 2015; Walker et al., 2015; Lee et al., 2018). The characteristics of these studies are shown in Table 1, and the complete version of it is enclosed in the Supplementary Material. The longest time period of the studies was from 1997 to 2012 (Capurso et al., 2013; Chiu et al., 2013; Funch et al., 2014; Lewis et al., 2015; Lin et al., 2015; Lu et al., 2015; Walker et al., 2015; Lee et al., 2018; Tseng, 2018; Lai et al., 2019; Feola et al., 2022). Of the 11 studies, six studies were based on the Western population, while the remaining five were based on the Asian population. The majority of the diabetic patients included in these studies were taking multiple hypoglycemic drugs for glycemic control, which was taken into consideration and adjusted for in six of the 11 studies.

**TABLE 1:** Characteristics of included studies assessing the risk of PC in diabetic patients using hypoglycemic agents.

Study, year, and region	Design and time	Agent^a^	Metformin AOR (95% CI)	SU AOR (95% CI)	TZD AOR (95% CI)	Insulin AOR (95% CI)	Covariate adjustment
Feola et al., 2022, Italy	C-c study, population-based, NR	1	0.28 (0.08–0.93)	NR	NR	NR	Age at diagnosis, sex, BMI, family history, alcohol use, smoking habits, comorbidities, obesity, hypertriglyceridemia, hypercholesterolemia, low HDL cholesterol, inflammatory bowel diseases, celiac disease, and pancreatitis
Lai et al., 2019, Taiwan, China	C-c study, population-based, 2000–2013	1	1.68 (0.84–3.34)	NR	NR	NR	Sex, age, other antidiabetic drug use, and comorbidities (alcohol-related diseases, biliary stone, cardiovascular disease, chronic obstructive pulmonary disease, and pancreatitis)
Lee et al., 2018, Korea	Cohort, 2009–2012	1, 2, 3, and 4	0.86 (0.77–0.96)	1.73 (1.57–1.91)	0.82 (0.68–0.98)	2.86 (1.43–5.74)	Age, sex, chronic pancreatitis, acute pancreatitis, hepatitis B, hepatitis C, biliary disease, alcoholism, non-alcoholic fatty liver disease, income of the lowest quartile, and different exposed antidiabetic medications
Tseng et al., 2018, Taiwan, China	Cohort, 1999–2005	1	0.49 (0.25–0.96)	NR	NR	NR	Age, sex, occupation, living region, obesity, tobacco abuse, *H. pylori*, comorbidities, and other medications
Lewis et al., 2015, United States of America	Cohort, 1997–2012	1, 2, 3, and 4	1.21 (1.02–1.43)	1.49 (1.22–1.81)	1.41 (1.16–1.71)	2.34 (1.97–2.78)	Age, prolonged use of other diabetes medications, year of cohort entry, sex, race–ethnicity, income, current smoking status, baseline HbA1c, DM duration, new DM diagnosis, creatinine, and congestive heart failure
Lu et al., 2015, United Kingdom	Cohort, 1996–2010	1, 2, 3, and 4	2.63 (1.99–3.46)	3.39 (2.54–4.54)	3.63 (2.33–5.68)	10.15 (5.95–17.32)	Age, sex, body mass index, smoking, alcohol consumption, Townsend deprivation index, and DM
Lin et al., 2015, Taiwan, China	Cohort, 2005–2010	2, 3, and 4	NR	1.77(0.70–4.50)	0.71 (0.30–1.70)	1.91 (0.88–4.15)	Sex, age, hypertension, dyslipidemia, obesity, gout, hepatitis B, hepatitis C, liver cirrhosis, and duration of ADT exposure
Walker et al., 2015, United States of America	C- c study, clinic-based, 2006–2011	1, 3, and 4	0.81 (0.42–1.58)	NR	0.80 (0.35–1.86)	1.48 (0.74–2.99)	Age, sex, and other classes of DM drugs
Funch et al., 2014, United States of America	Cohort, 2010–2013	1, 2, and 3	0.81 (0.32–2.05)	0.40 (0.15–1.06)	0.49 (0.17–1.41)	NR	Age, gender, healthcare utilization, and DM Complications and Severity Index
Chiu et al., 2013, Taiwan, China	Cohort, 2000–2007	1, 2, 3, and 4	1.12 (0.63–2.00)	2.36 (1.21–4.61)	1.08 (0.52–2.25)	1.67 (1.00–2.79)	Age, sex, and selected comorbidities
Capurso et al., 2013, Italy	C- c study, hospital-based, 2010–2011	4	NR	NR	NR	6.03 (1.74–20.84)	Age, sex, and selected covariates

AOR, adjusted odds ratio; CI, confidence interval; SU, sulfonylurea; TZDs, thiazolidinediones; NR, not reported; C-c study, case–control study; *H. pylori*, *Helicobacter pylori*.

^a^ The numbers stand for 1—meformin, 2—sulfonylureas, 3—TZDs, and 4—insulin.

The Newcastle–Ottawa quality score for observational studies ranges from 6 to 9, and the median quality score is 8. The methodological quality of all studies is depicted in Table 2 and Table 3. In addition, according to the NOS criteria, scores 1–3, 4–6, and 7–9 are defined as low, medium, and high quality, respectively. The overall methodological quality of the studies was moderate to high.

**TABLE 2:** Methodological quality of cohort studies included in this meta-analysis.

Ref.	Representativeness of the exposed cohort	Selection of the non-exposed cohort	Ascertainment of exposure	Demonstration of outcome of interest not present at the start of the study	Control for important factors^a^	Assessment of outcome	Follow-up long enough for outcomes to occur	Adequacy of follow-up of cohorts	Total quality scores
Lee et al. (2018)	*	*	*	*	**	*	*	*	9
Tseng (2018)	*	*	*	*	-	*	*	*	8
Lewis et al. (2015)	*	*	*	*	**	*	*	*	9
Lu et al. (2015)	*	*	*	*	**	*	*	*	9
Lin et al. (2015)	-	-	*	*	**	*	*	*	7
Funch et al. (2014)	*	*	*	*	**	*	*	*	9
Chiu et al. (2013)	*	*	*	*	**	*	*	*	9

^a^ A maximum of two stars could be awarded for this item. Studies that controlled for age and sex received one star, whereas studies that controlled for additional covariants received another star.

**TABLE 3:** Methodological quality of c-c studies included in this meta-analysis.

Ref.	Adequate definition of cases	Representativeness of cases	Selection of controls	Definition of controls	Control for important factors^a^	Exposure assessment	Same method of ascertainment for cases and cohorts	Non-response rate	Total quality scores
Feola et al. (2022)	-	-	*	-	**	*	*	*	6
Lai et al. (2019)	-	*	*	*	**	-	*	*	7
Walker et al. (2015)	*	*	-	*	**	*	*	*	8
Capurso et al. (2013)	*	*	*	*	**	*	*	*	9

^a^ A maximum of two stars could be awarded for this item. Studies that controlled for age and sex received one star, whereas studies that controlled for additional covariants received another star.

### Hypoglycemic agents and PC incidence

#### Metformin and PC incidence

**FIGURE 2:**
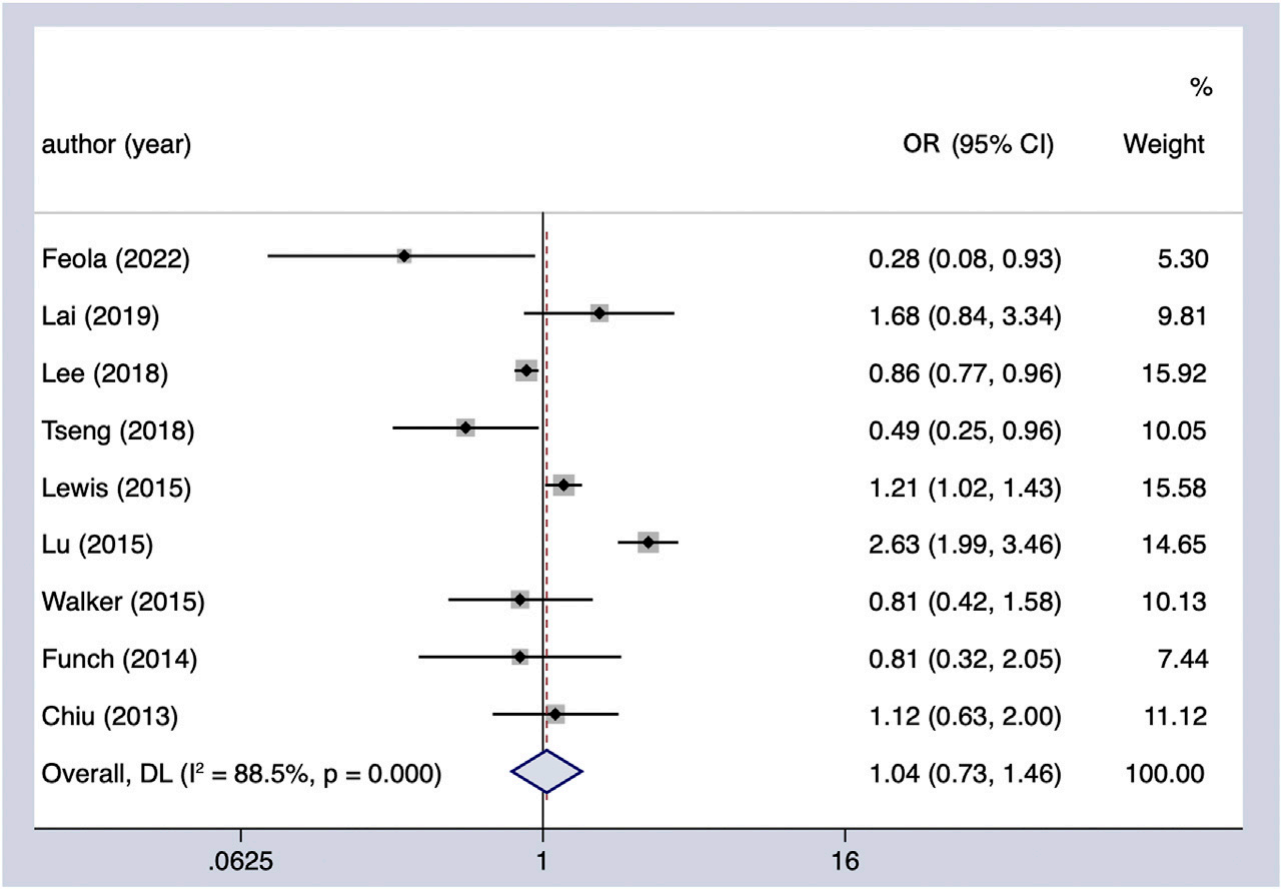
Summary of the adjusted odds ratios of observational studies assessing the association between the incidence of PC and metformin use in diabetic patients. CI, confidence interval.

Meta-analysis of nine observational studies that reported the risk of PC associated with metformin intake in patients with DM demonstrated no significant protective or harmful effect (OR = 1.04, 95% CI 0.73–1.46) (Figure 2). There was significant heterogeneity among the analyzed studies (Cochran’s Q test *p* < 0.01, I^2^ = 86%), and it could not be explained by study design, region, or whether adjusted for other hypoglycemic agents in the subgroup analysis we conducted (Table 4). Furthermore, we excluded the individual study to assess whether a specific study affected the result in the sensitivity analysis, and the findings of the primary analysis did not alter.

**TABLE 4:** Subgroup analyses.

Subgroup	N	Random-effects model		Heterogeneity between groups (P)^a^
		OR	95% CI	
*Metformin*				
***All observational studies***	9	1.04	0.73–1.46	
*Study design*				
Cohort	6	1.10	0.74–1.65	0.541
Case–control	3	0.82	0.34–1.94	
*Region*				
Asian	4	0.92	0.64–1.33	0.658
Western	5	1.08	0.61–1.89	
*Adjusted for other hypoglycemic agents*				
Yes	5	0.96	0.72–1.27	0.871
No	4	1.03	0.44–2.44	
***Sulfonylurea***				
***All observational studies***	6	1.79	1.29–2.49	
*Region*				
Asian	3	1.74	1.58–1.92	0.671
Western	3	1.46	0.66–3.24	
*Adjusted for other hypoglycemic agents*				
Yes	3	1.68	1.54–1.83	0.945
No	3	1.62	0.57–4.58	
***TZDs***				
***All observational studies***	7	1.13	0.73–1.75	
*Study design*				
Cohort	6	1.18	0.73–1.90	0.431
Case–control	1	0.80	0.35–1.84	
*Region*				
Asian	3	0.83	0.70–0.99	0.193
Western	4	1.34	0.66–2.70	
*Adjusted for other hypoglycemic agents*				
Yes	4	0.97	0.64–1.46	0.614
No	3	1.34	0.41–4.33	
*Insulin*				
***All observational studies***	7	2.85	1.75–4.64	
*Study design*				
Cohort	5	2.95	1.64–5.28	0.912
Case–control	2	2.71	0.69–10.59	
*Region*				
Asian	3	1.99	1.39–2.87	0.200
Western	4	3.69	1.55–8.77	
*Adjusted for other hypoglycemic agents*				
Yes	4	2.29	1.95–2.68	0.310
No	3	4.58	1.21–17.33	

CI, confidence interval; OR, odds ratio.

^a^ If *p* < 0.10, then it implies that the difference between the two subgroups is significant and may explain the heterogeneity observed in the overall analysis.

#### Sulfonylureas and PC incidence

**FIGURE 3:**
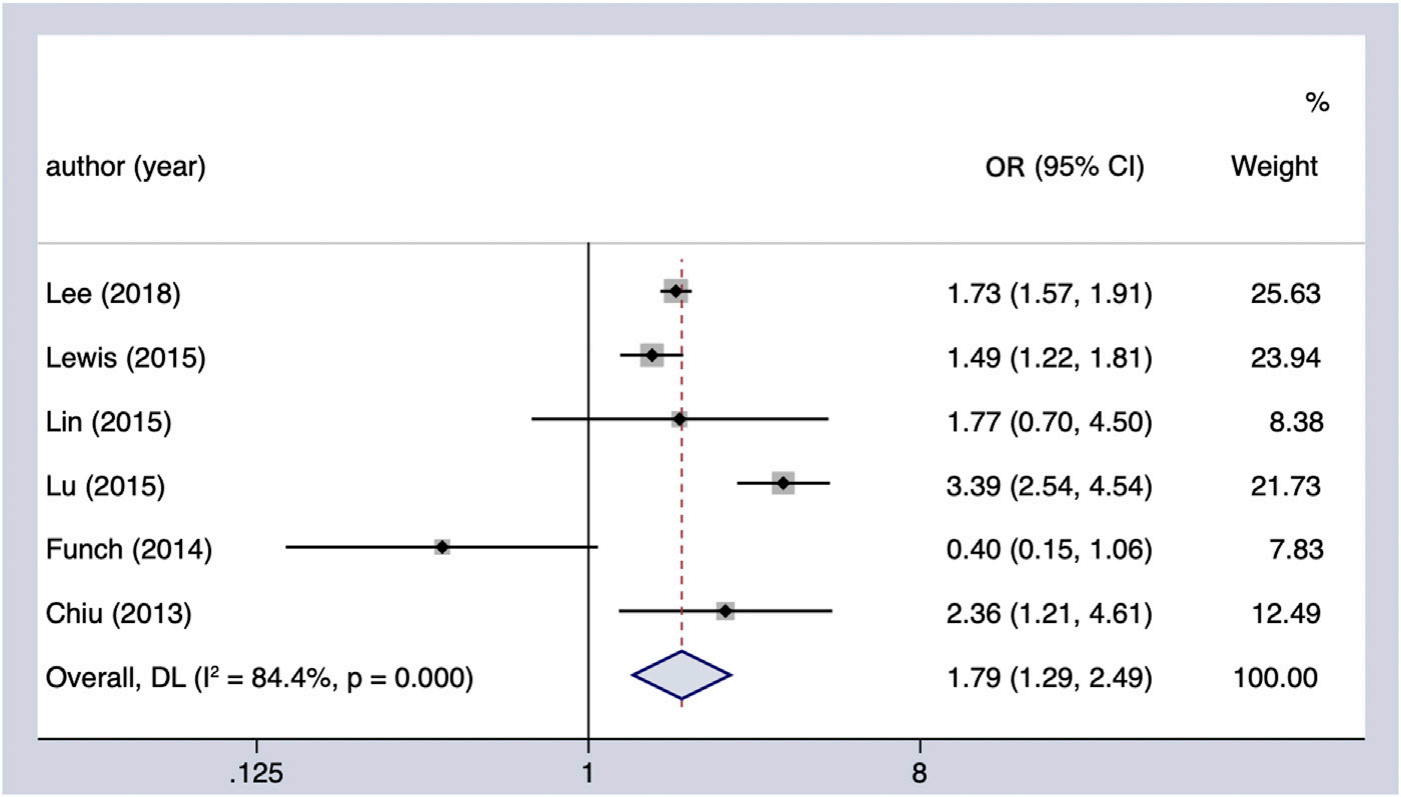
Summary of the adjusted odds ratios of observational studies assessing the association between the incidence of PC and sulfonylurea use in diabetic patients. CI, confidence interval.

The meta-analysis of six observational studies showed that SU administration increased the PC incidence by 79% (OR = 1.79, 95% CI 1.29–2.49), which was statistically significant (Figure 3). The analysis demonstrated considerable heterogeneity across studies (Cochran’s Q test *p* < 0.01, I^2^ = 84.4%), which could not be fully explained by the subgroup analysis (Table 4). The results remained unchanged upon restricting the analysis to studies that considered the concomitant effect of other hypoglycemic agents (OR = 1.68, 95% CI 1.54–1.83), and the subgroup analysis based on region indicated that SU may play an oncogenic role in the Asian population (OR = 1.74, 95% CI 1.58–1.92). We performed sensitivity analysis by leaving out the individual study, and the results were robust.

#### Thiazolidinediones and PC incidence

**FIGURE 4:**
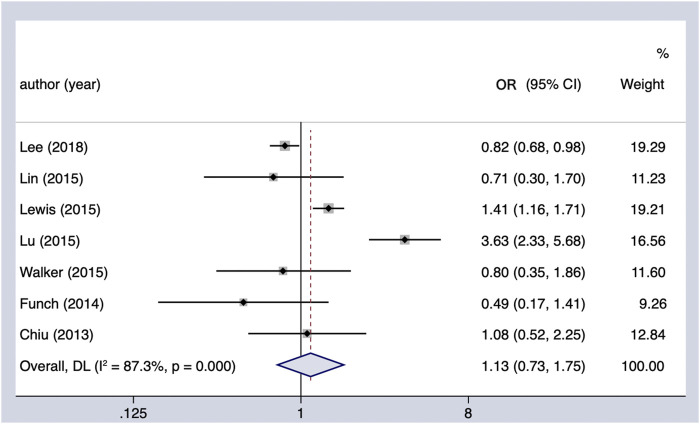
Summary of the adjusted odds ratios of observational studies assessing the association between the incidence of PC and TZD use in diabetic patients. CI, confidence interval.

The use of TZDs (as compared with the non-use of TZDs) was not associated with a significant increase in the risk of PC in patients with DM (OR = 1.13, 95% CI 0.73–1.75) (Figure 4). Considerable heterogeneity was observed across the seven studies included (Cochran’s Q test *p* < 0.01, I^2^ = 87.3%), and it could not be fully explained by the factors in the subgroup analysis (Table 4). However, when it comes to region, it showed a possible protective effect on the Asian population (OR = 0.83, 95% CI 0.70–0.99). In the sensitivity analysis, when we excluded the individual study, the findings of the primary analysis remained the same.

#### Insulin and PC incidence

**FIGURE 5:**
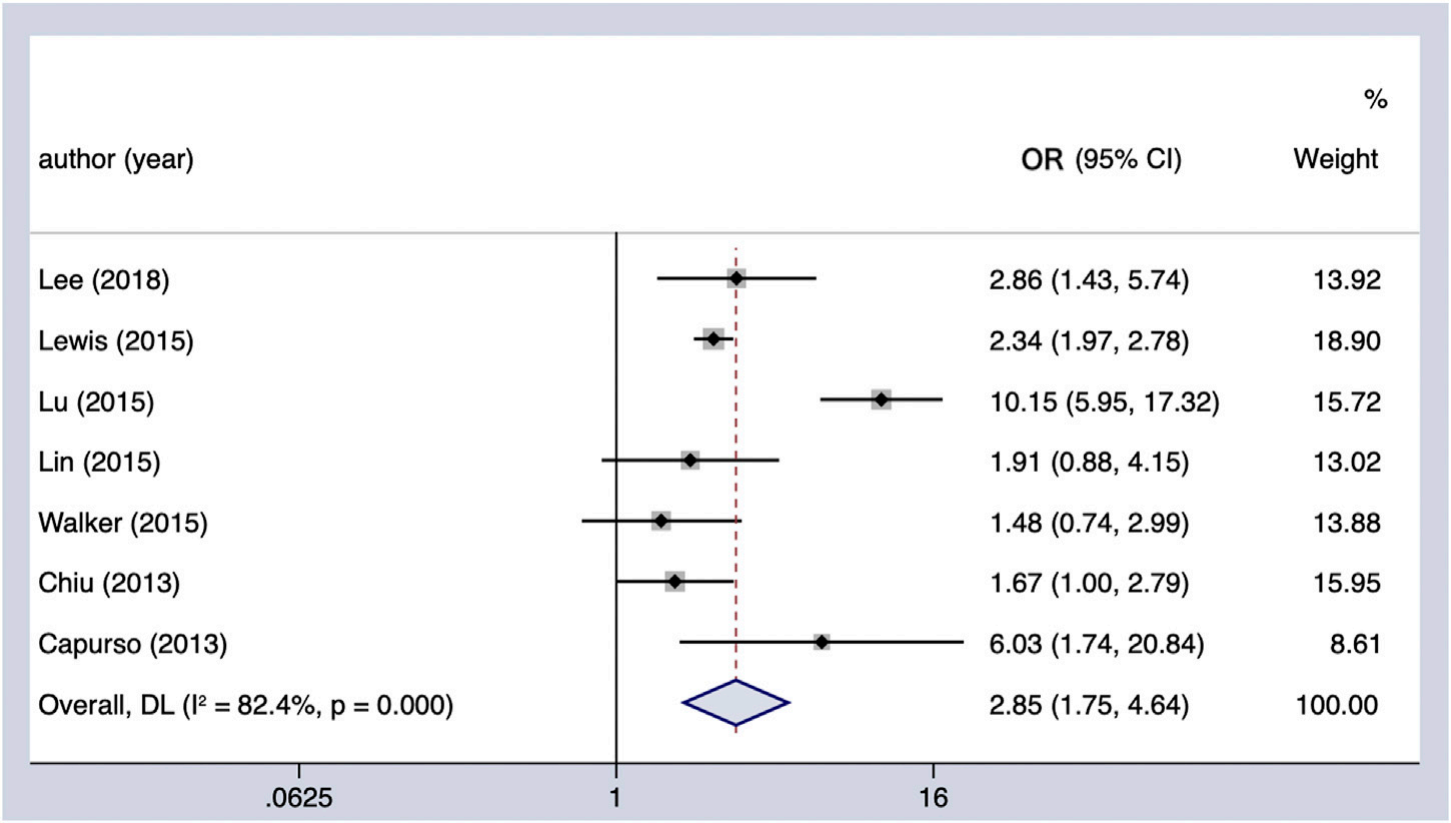
Summary of the adjusted odds ratios of observational studies assessing the association between the incidence of PC and insulin use in diabetic patients. CI, confidence interval.

Meta-analysis of all observational studies demonstrated that the use of insulin was associated with a statistically significant 185% increase in the risk of PC in patients with DM (OR = 2.85, 95% CI 1.75–4.64) (Figure 5). Considerable heterogeneity was found across the seven observational studies (Cochran’s Q test *p* < 0.01, I^2^ = 82.4%), and it could not be completely explained by the subgroup analysis we conducted (Table 4). However, it is noteworthy that the oncogenic effect on the Western population (OR = 4.58, 95% CI 1.21–17.33) was much worse than that on the Asian population (OR = 2.29, 95% CI 1.95–2.68). Furthermore, the results remained the same when restricting the analysis to studies that considered the concomitant effect of other hypoglycemic agents. The results were unchanged in sensitivity analysis when removing the studies included one by one.

#### Publication bias

There was no indication of significant publication bias both quantitatively (*p* = 0.860 for metformin, *p* = 0.986 for SU, *p* = 0.954 for TZDs, and *p* = 0.611 for insulin; *p* < 0.10 was considered to indicate significant publication bias) and qualitatively on visual inspection of the funnel plot.

## Discussion

In our comprehensive meta-analysis of 11 studies assessing the influence of conventional hypoglycemic agents on PC risk of patients with DM, the results showed that metformin intake in patients with DM has no significant protective or harmful effect (OR = 1.04, 95% CI 0.73–1.46), while SU administration increased the PC incidence by 79% (OR = 1.79, 95% CI 1.29–2.49). TZDs were not associated with a significant increase in the risk of PC in patients with DM (OR = 1.13, 95% CI 0.73–1.75), and the use of insulin was associated with a statistically significant 185% increase in the risk of PC in patients with DM (OR = 2.85, 95% CI 1.75–4.64).

We found that, in comparison with non-use, the use of metformin and TZDs had no statistically important associations with the incidence of PC in diabetic patients, while the use of SU and insulin was associated with an increase of 79% and 185% in the risk of PC, respectively. The results were stable across both cohort and case–control studies and persisted after adjusting for the concomitant effect of other hypoglycemic agents, whereas significant heterogeneity was observed across studies, which limited the meta-analysis. In addition, the subgroup analysis showed that SU may have an oncogenic effect, especially on the Asian population and those adjusted for other hypoglycemic agents. Apart from that, it also indicated that TZDs were associated with the protective effect on the Asian population, and the use of insulin was shown to have a greater oncogenic effect on the Western population in cohort studies and those not adjusted for other hypoglycemic agents.

The strength of this meta-analysis is that it assessed the comprehensive and simultaneous effects of all conventional hypoglycemic agents on the risk modification of PC. Although Singh et al. (2013) conducted a similar meta-analysis on the risk of PC in patients with DM, it was almost 10 years ago, and the change in lifestyle and human disease spectrum is considerable. Some recent meta-analyses studied the associations between only one or two hypoglycemic agents and the risk of PC (Soranna et al., 2012; Wang et al., 2014), and most meta-analyses focused on the relationships between hypoglycemic agents and the survival or prognostic significance of PC patients (Zhou et al., 2017; Jian-Yu et al., 2018; Wan et al., 2018).

Metformin is one of the most commonly used drugs for the treatment of DM. In recent years, some clinical studies have found that in addition to lowering blood glucose levels, metformin also has certain effects on alleviating fatty liver, protecting the cardiovascular system, inhibiting tumor growth, and relieving symptoms of neurodegenerative diseases (Morales and Morris, 2015; Barzilai et al., 2016; Rena et al., 2017; Jian-Yu et al., 2018; Foretz et al., 2019; Lv and Guo, 2020). It is generally believed that metformin acts directly on the liver, kidney, and intestine. After being transported into cells, metformin plays important biological roles in reducing fat content and lowering blood glucose levels mainly by activating the adenosine monophosphate-activated protein kinase (AMPK) signaling pathway (Rena et al., 2017). However, the direct molecular target of metformin’s action has long been unclear. Previous studies have shown that metformin acts through the classical AMP-dependent activation of AMPK by inhibiting mitochondrial electron transport chain complex 1, which increases AMP levels (Foretz et al., 2014; Rena et al., 2017). However, the concentration of metformin used in the trials was much higher than that of the clinical agent in all *in vitro* experiments, so this signaling pathway cannot explain the actual effect of the clinical drug benefit. Ma et al. (2022) found the direct target of metformin, PEN2 (γ-secretase), and elucidated the molecular biological mechanism of its biological effect by binding to the ATP6AP1 subunit of transmembrane protein v-ATPase to activate the lysosome AMPK pathway. TZDs do not stimulate insulin secretion, but they increase the sensitivity of peripheral tissues to insulin, especially those that are targets of insulin action: skeletal muscle, liver, and adipose tissue, thereby increasing the utilization of glucose in muscle, reducing the production of endogenous glucose in the liver, promoting the synthesis of fat, and inhibiting its decomposition so that the metabolic disorders in the body tend to be normal and indirectly achieve the effect of lowering the glycemic level (Nanjan et al., 2018). In another meta-analysis (Wang et al., 2014), the result showed that metformin reduces the risk of PC in patients with DM, and there are studies proving that TZDs inhibit the proliferation and metastasis of human pancreatic cancer cells (Ninomiya et al., 2014). However, this study did not find statistically significant results about metformin and TZDs, which may be explained by the considerable heterogeneity observed across the included studies, and it can also be caused by the simultaneous effect of other hypoglycemic agents.

SU act on K^+^ channels in islet beta cells to induce their closure, resulting in increased fasting and postprandial insulin levels, which is oncogenic.

For insulin, it promotes the development of PC through the following biological effects: promoting cell proliferation and growth, activating the RTK signal pathway, and promoting angiogenesis (Chang et al., 2002; Moschos and Mantzoros, 2002; Boyd, 2003; Leinonen et al., 2003; Mauro et al., 2003; Tamakoshi et al., 2003). Karlstad et al. (But et al., 2017) found that in addition to the reduced risk of prostate cancer, the risks of liver cancer, PC, kidney cancer, gastric cancer, and respiratory cancer were significantly increased in diabetic patients using insulin. Insulin glargine users had a reduced risk of colon cancer and an increased risk of breast cancer. Studies have shown an increased risk of pancreatic and prostate cancer in insulin glargine users (Colmers et al., 2012). Other studies have shown that insulin glargine does not increase the risk of cancer in users (Chang et al., 2011; Du et al., 2012; Tang et al., 2012). The aforementioned studies have limitations, such as short follow-up time, and the conclusions are not very reliable.

We found a significant oncogenic effect of SU on the Asian population (OR = 1.74, 95% CI 1.58–1.92), which was not observed in the Western population (OR = 1.46, 95% CI 0.66–3.24). In addition, for TZDs, a statistically significant protective effect on the Asian population was observed (OR = 0.83, 95% CI 0.70–0.99). For insulin users, the oncogenic effect is 170% greater in the Western population than that in the Asian population. The regional discrepancy may be explained by ethnic differences, environmental factors, the gap in economic development, and different lifestyles, particularly in dietary habits. The Western diet (WD) is an unhealthy diet high in fat and characterized by binge eating, frequent snacking, and a prolonged postprandial state. Studies have shown that the Western diet leads to hyperinsulinemia, insulin resistance, dyslipidemia, and hyperactivity of the sympathetic nervous system and renin–angiotensin system (Malesza et al., 2021). The Asian dietary pattern also has many carcinogenic factors, such as excessive consumption of pickled food, insufficient intake of fresh vegetables and fruits due to the small amount of arable land in countries such as Korea and Japan, and the high rate of *Helicobacter pylori* infection because Asian people are not used to serving of individual dishes. It can be seen that the regional discrepancy is caused by the combined influence of ethnic differences, environmental factors, and different lifestyles.

Apart from that, most diabetic patients in the studies were simultaneously taking multiple hypoglycemic agents, which may influence the result, and this should not be neglected. For example, a patient in the “on metformin” group was on metformin and insulin medication at the same time, and metformin plays a neutral role in cancer risk, while insulin has a great oncogenic effect; the result may be magnified by the effect of insulin. In addition, the age of the patients should be considered. Aged patients tend to use insulin more, and the control of glycemic levels is relatively poor. Furthermore, elderly diabetic patients have more complications and concomitant diseases, thus causing polypharmacy. This may cause the potential overestimate of the oncogenic effect of insulin and the great heterogeneity seen in the study.

As known, adherence and persistence to antidiabetic drugs are very low. This aspect could have contributed to the high risk of PC in diabetic patients. In other words, the higher risk of PC in patients receiving SU and insulin, in part, can be a result of non-adherence to the antidiabetic medication and, therefore, a direct consequence of T2D progression. Indeed, T2D itself is one of the major risk factors for developing PC.

This meta-analysis has several limitations. First, the study only included observational studies because there was no relevant published RCT from 2012 to 2022, which may affect our results. Second, most studies assessed the cancer incidence caused by the use of more than one hypoglycemic agent, and the simultaneous effect of other agents was not excluded, which may modify the cancer risk. Third, the included studies were mainly conducted in East Asia and North America, while only one study was conducted in Italy and Europe, and none in other continents, which limited our study. Finally, the adjusted variables varied among studies, and many of them were insufficient, which may cause considerable heterogeneity and influence the results.

In conclusion, SU and insulin may modify the incidence of PC in diabetic patients, and the effect of hypoglycemic agents may vary in regions (Asian and Western). There was considerable heterogeneity across studies, and the relationship between hypoglycemic agents and the incidence of PC in diabetic patients should be further interpreted with well-adjusted data and better-organized clinical trials.

## Data Availability

The original contributions presented in the study are included in the article/Supplementary Material; further inquiries can be directed to the corresponding author.
